# Design and implementation of an online admissions interview for selection to nursing and midwifery programmes: a partnership approach

**DOI:** 10.1186/s12912-022-01058-y

**Published:** 2022-10-17

**Authors:** Marian Traynor, Stephanie Dunleavy, Sonja McIlfatrick, Donna Fitzsimons, Michael Stevenson, Roisin McEvoy, Caroline Mulvenna

**Affiliations:** 1 Faculty of Medicine Health & Life Sciences, Queen’s University Belfast, 90 Lisburn Road,, BT9 6AG Belfast, Northern Ireland; 2 School of Nursing, Ulster University, Shore Road,, BT37 OQB Newtownabbey, Co Antrim UK; 3 School of Nursing & Midwifery Medical Biology Centre, Queen’s University Belfast, 97 Lisburn Road, BT9 7BL Belfast, Northern Ireland; 4 Queen’s University Belfast, Whitla Medical Building, 97 Lisburn Road, Belfast, Northern Ireland; 5 Queen’s University Belfast, BT9 6AG Belfast, Northern Ireland

**Keywords:** Undergraduate nursing, Undergraduate midwifery, Staff workload, Selection, Virtual interviews

## Abstract

**Background:**

The recent surge in applications to nursing in the United Kingdom together with the shift towards providing virtual interviews through the use of video platforms has provided an opportunity to review selection methodologies to meet a new set of challenges. However there remains the requirement to use selection methods which are evidence-based valid and reliable even under these new challenges.

**Method:**

This paper reports an evaluation study of applicants to nursing and midwifery and reports on how to plan and use online interviews for in excess of 3000 applicants to two schools of nursing in Northern Ireland. Data is reported from Participants, Assessors and Administrators who were asked to complete an online evaluation using Microsoft Forms.

**Results:**

A total of 1559 participants completed the questionnaire. The majority were aged 17–20. The findings provide evidence to support the validity and reliability of the online interview process. Importantly the paper reports on the design and implementation of a fully remote online interview process that involved a collaboration with two schools of nursing without compromising the rigour of the admissions process. The paper provides practical, quantitative, and qualitative reasons for concluding that the online remote selection process generated reliable data to support its use in the selection of candidates to nursing and midwifery.

**Conclusion:**

There are significant challenges in moving to online interviews and the paper discusses the challenges and reflects on some of the broader issues associated with selection to nursing and midwifery. The aim of the paper is to provide a platform for discussion amongst other nursing schools who might be considering major changes to their admissions processes.

## Introduction

This paper reports on the design, implementation and evaluation of an online admissions interview for selection to nursing and midwifery across two schools of nursing in two universities in Northern Ireland. The rationale for a shared selection process across the two universities had previously been discussed by senior academics and the Chief Nursing Officer (CNO) pre-pandemic, however Covid-19 provided the impetus to implement the proposed changes within a more immediate timescale. The paper provides a brief overview of the purposes and processes for selection to nursing and midwifery. It also provides the context for the planning and implementation of an interview process with two universities that involved in excess of 3,000 applications to two schools of nursing. Finally, the evaluations from all the key stakeholders involved in the process will be discussed as well as lessons learned that may be helpful to other schools who may be considering adding an interview to their selection processes.

## Background

In Northern Ireland (NI), undergraduate nursing places are commissioned by the Department of Health NI and both universities had seen an increase year on year in the number of commissioned places. This is reflective of what is happening across the UK and also globally and is a direct result of policies to promote the recruitment and retention of qualified nurses. According to predictions by WHO the world will need an additional 9 million nurses and midwives by the year 2030 (WHO 2020) [10]. 2021 saw a significant increase in applications to nursing courses across the UK as a result of the effect of the pandemic (Nursing Times, 2021) [6] and this has also been the case in NI.

There is already work in progress to increase nursing student numbers globally (Drennan and Ross 2019) [3]. Recent data published by the American Association of Colleges of Nursing (AACN, 2021) [1] showed that enrolment increased by 5.6% with 251,145 students undertaking nursing programmes in America. Similarly figures from the Australian Government Department of Health (2021) [2] indicate a 3.8% growth in the nursing and midwifery workforce with an expectation that this will grow further over the next decade as Australia’s population changes.

The increase in commissioned places in pre-registration nursing courses by the Department of Health Northern Ireland (NI) therefore reflects the urgent changes to workforce planning more generally. A review of the nursing and midwifery workforce in 2009 underestimated the number of undergraduate nursing places required in NI. This resulted in the Department of Health (NI) significantly increasing the commissioned nursing training places, with a total of 3,895 places commissioned between 2017 and 18 and 2020-21 (an annual average of 974 places). In 2020-21 an all-time high level of 1,210 places were commissioned and for some courses this represented a 49% increase in student places. This resulted in a significant number of applicants applying for two or more fields of nursing and/or midwifery for each commissioned place. Furthermore, as many as 50% of applicants were applying to both universities, this essentially meant that both universities were considering applications from the same group of approximately 1400 applicants every year. Applicants who met the selection criteria would be offered an interview for each university.

The aim of selection in pre-registration nursing and midwifery in the UK is to ensure that applicants are selected who will be successful and complete the three-year nursing programme, register with the Nursing and Midwifery Council (NMC) as competent practitioners and ultimately join the nursing and midwifery workforce. Selecting the best candidates is typically on the basis of two domains: academic ability and non-academic attributes considered desirable in a nurse or midwife (Traynor et al. 2017 [9]; NMC 2017 [5]; Cleland et al. 2012 [4]). Normally, academic ability is assessed based on secondary school examinations (or their equivalent) and this information is then used in conjunction with a personal statement to determine applicants who will be invited to interview.

Previously both universities used a face to face interview to determine the non-academic attributes. This was consistent with most UK nurse education providers however the significant difference was that one university used a multiple mini interview (MMI) where applicants rotate around seven short interviews and the other a more traditional 2:1 interview format with two assessors interviewing one applicant. Importantly both interview formats used a values-based approach that measured personal qualities such as motivation, empathy, integrity etc., (Gateway to Nursing, Northern Ireland Practice and Education Council for Nursing and Midwifery, 2014 [7]). Another significant factor was that both universities made the decision to remove the Personal Statement (PS) from the selection process for 2021 on the findings of a joint research initiative, led by the author of this paper, that determined that there were significant shortfalls with using the PS as a selection tool (Traynor and Barr et al. 2018)[8]. This changed the landscape significantly in that all applicants who demonstrated that they either had or could meet the academic entry requirements would ultimately be invited to interview. The other important decision taken by both universities was that as a consequence of the COVID-19 pandemic interviews would move to an online platform with the implementation date for a regional approach being moved forward to 2021. The challenge was not only to develop and implement a new regional interview process but to do so within in a five-month window.

## Methods

The Chief Nursing Officer for Northern Ireland established a Regional Admissions Group with representation from both universities, service users and students to manage the implementation of the new interview process that was to be piloted with the 2021 applicants to nursing and midwifery. This was chaired by the Heads of Schools of nursing from each university and a Task and Finish Group was subsequently established to implement the pilot project. The study did not require ethical approval as it was an agreed change to an admission process by the Chief Nursing Officer for Northern Ireland.

The Task & Finish Group had the following specific objectives:

1. To agree an online platform for the regional pilot study.
2. To agree the number and nature of the interview questions.
3. To organize staff training for the new regional approach.

### The project

#### Aims

The aim of the project was to design, implement and evaluate evidence for a shared interview process as a potential selection method for the recruitment of Nursing and Midwifery students that have attributes and behaviours suited to nursing and midwifery. This paper will focus on the design, implementation and evaluation from all the key stakeholders: the candidates; the assessors (academics, practice colleagues, service users) and the admission staff.

#### Design

The project design was a cross-sectional evaluation study. The participants were applicants to nursing and midwifery pre-registration programmes, the assessors were either academic staff, practice staff or service users. The interview questions were reviewed by members of a Task and Finish group that was made up of academic staff, admissions staff, service users and students and cross referenced against the NMC competencies and attributes that are valued to support a career in nursing (Nursing and Midwifery Council (NMC) 2018) and also the attributes valued to realize future potential for a career in nursing published by the Northern Ireland Practice and Education Council for Nursing and Midwifery (NIPEC), in 2014[7].

A 12-item questionnaire, incorporating three Likert scales, plus one yes/no answer, and two demographic questions (in order to construct profiles of the population), one on field of nursing and university applied to (in order to establish duplicate applications), one on digital skills (in order to establish entrance criteria for digital competence) was used to collect data on the candidates. A five-point scale consisting of “strongly agree”, “agree”, “undecided”, “disagree, “strongly disagree” was utilised. The questionnaire also had four qualitative “free response” questions to allow candidates to elaborate, expand, clarify or illustrate their answers. To ensure that the questions were appropriate the questionnaires were submitted to members of the Regional Admission group to review and agree relevance of the questions. The questionnaire was embedded in Microsoft Forms and candidates were provided with a link to the questionnaire after they had completed the online interview. The analysis was completed using simple descriptive statistics.

The questionnaire for the assessors was made up of twelve questions that included one Likert scale, plus one multiple choice question with explanation of answer, and five demographic questions (in order to construct profiles of the population).

A five-point scale consisting of “strongly agree”, “agree”, “undecided”, “disagree, “strongly disagree” was utilised. The questionnaire also had five qualitative “free response” questions to allow candidates to elaborate, expand, clarify or illustrate their answers and provide any further comments.

The evaluation questionnaire for Admission staff consisted of five questions; two had yes/no answers and three were designed to capture areas for improvement.

To ensure that the questionnaires were appropriate they were submitted to members of the Regional Admission group to review and agree relevance of the questions. No changes were requested. The questionnaires were subsequently embedded in Microsoft Forms and participants were provided with a link to the relevant questionnaire.

#### Participants

All applicants to Nursing and Midwifery participated in the Regional Interview (n = 2664). Applicants were processed through the normal Admissions routes for both universities. The design and format of the new regional approach was available on the webpages for both universities and both schools of nursing had information on the new regional approach. Information was also shared with career teachers at institutional career teacher events. This included the need for the changes in the selection process, confirmation that the personal statement was no longer being used as selection for invitation to interview and detail about the online interview platform and format that was being used. Each applicant was also requested to give consent to any sharing of information across both universities for the purposes of the selection interview.

The interview process took place over a 10-week period. A total of 170 assessors participated in the regional approach made up of: 136 academics; 13 service users; 21 colleagues from clinical practice. In order to maintain the integrity and security of the questions all candidates and assessors were required to sign a confidentiality agreement.

#### Data collection

The following sources were used to evaluate the regional process:

- Candidates’ scores from the four questions;
- Candidates’ responses to the evaluation questionnaire.
- Candidates’ responses to the evaluation of the online platform.
- Assessors’ response to the evaluation questionnaire.
- Admissions staff responses to the evaluation questionnaire.

### Regional interview online platform

The first task was to source an online platform that could deal with significant numbers and importantly permit the asynchronous marking of the video by assessors who would be both internal and external to the university. An online platform currently used within one HEI was reviewed and was considered to fit the requirements of the regional interview process. The company was advised of the pilot nature of the process and after providing a demonstration of the software, were appointed by the Regional Admissions Group.

### Regional interview questions

The next task was to find agreement on the interview format and it was determined that a values-based approach based on the Gateway values, familiar to and already used by both universities, should be the starting point. This resulted in an interview format that used four short scenario-based questions, in a timed period, to obtain a score of each applicant’s performance on scenarios measuring six values: trust, integrity, commitment to personal development, accountability, person centredness and team work.

The questions were designed to test specific values and attributes such as communication, empathy, critical thinking and ethics and were mapped to align with the Gateway values as outlined in Table 1.

**Table 1:** Sample of Questions mapped to Gateway Values (2014)

	GATEWAY VALUES					
**Questions**	**TRUST**	**INTEGRITY**	**ACCOUNTABILITY**	**PERSONAL DEVELOPMENT**	**PERSON CENTREDNESS**	**TEAM WORKING**
1 The Shoplifter	**x**		**x**		**x**	
2 – The Celebration		**x**	**x**			**x**
3 – The Drug Dealer	**x**		**x**		**x**	
4 – The Assignment	**x**	**x**	**x**			
5 – Alternative Therapies	**x**			**x**	**x**	
6 – Social Media	**x**	**x**			**x**	
7 – The Homeless Person	**x**	**x**			**X**	
8 – The Photos	**x**	**x**			**x**	

One question focused on reasons why the applicant had applied to nursing/midwifery.

Applicants had 1.5 min to read each of the four question and 3 min to answer each question. The total time for this activity was 18 min. Questions were taken from a previously validated bank of questions in both universities.

### Assessors

One hundred and seventy assessors from the two universities participated in the interview process. Assessors were service users, practice partners and academics and were randomly paired by the administrators for each university, so that each candidate was assessed independently by two assessors. On average each assessor reviewed 30 videos. In preparation for the role of assessor all assessors were invited to participate in a live information and Q&A session prior to assessing the video interviews. This session was also recorded so that assessors could revisit any aspect of the session and it was delivered jointly by both Universities.

### Marking of the questions

Scoring sheets previously developed and validated by one university were used; these were adapted to Microsoft Forms and once completed were automatically uploaded to a central file. The assessors were required to mark each value separately (for example, for question 1, the values to be assessed were trust, accountability and person centeredness). These were scored from ‘weak’ to ‘excellent’ with a range of 1–6. Also, to provide a separate global score, descriptors were provided through a global score sheet as a guide for the assessors to indicate their overall impression of those values displayed by the candidate. Global scores ranged, again, from 1 to 6 with descriptors ranging from ‘weak’ to ‘excellent’.

### Preparation of participants

All applicants to both universities were enrolled by Admissions staff to the online Platform. Once enrolled they had access to information on the interview process as well as specific information on how to prepare for the interview. Importantly they were also able to undertake as many practice attempts as they wished before actually participating in the interview.

### Preparation of assessors

All assessors were invited to an online training event where the specific details around the new regional approach were discussed and specific information on the scoring of candidates was provided. The session was recorded and made available to assessors.

## Results

### Scores from the interview questions

The four questions taken by a student were assessed by the same pair of assessors and global scores were applied only on the basis of the impression the candidate gave when assessed across the four questions. This permitted global cut-scores to be estimated - but based upon a combination of four questions rather than individual questions. Effectively therefore every interview combination had a unique cut-score.

Each interview combination was also assessed for reliability by Cronbach’s alpha. Cronbach’s alpha is a measure of internal consistency. An overall alpha value was set by calculating a weighted mean across all combinations. Using this, with the overall unadjusted standard deviation of interview scores across all candidates, the standard error of the measurement was struck and this value added to the required cut-score for every candidate.

Each candidate had their interview score adjusted according to their particular cut-score and hence a single ranked list of participants was formed.

### Evaluation by stakeholders

Microsoft Forms automatically generated pie chart and bar graphs to summarise the results from both the candidates and the assessors and is depicted in the figures below. Qualitative data was also captured via Microsoft Forms.

### The candidates

**Fig. 1:**
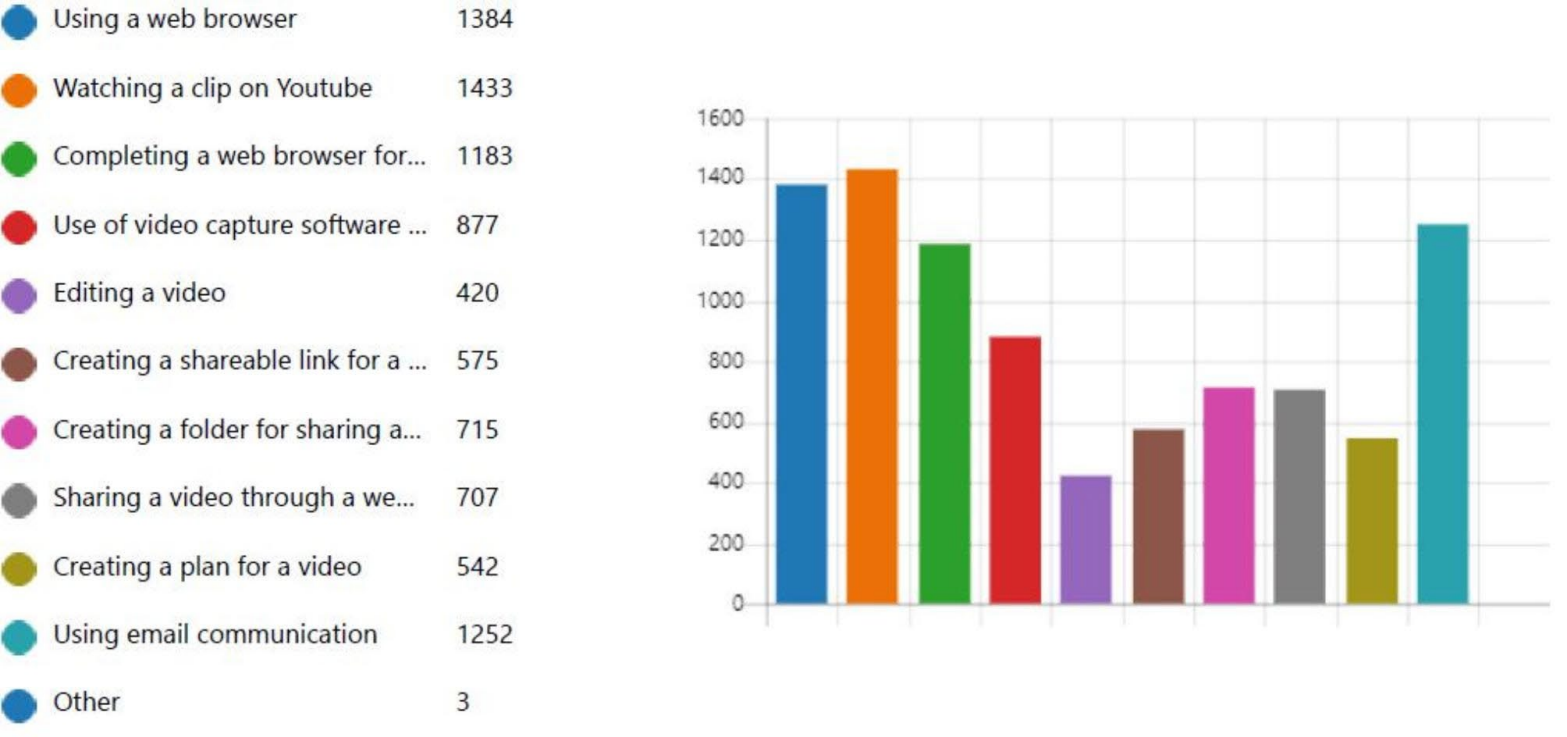
Proficiency in digital literacy skills BEFORE undertaking video interview (tick all that apply)

**Fig. 2:**
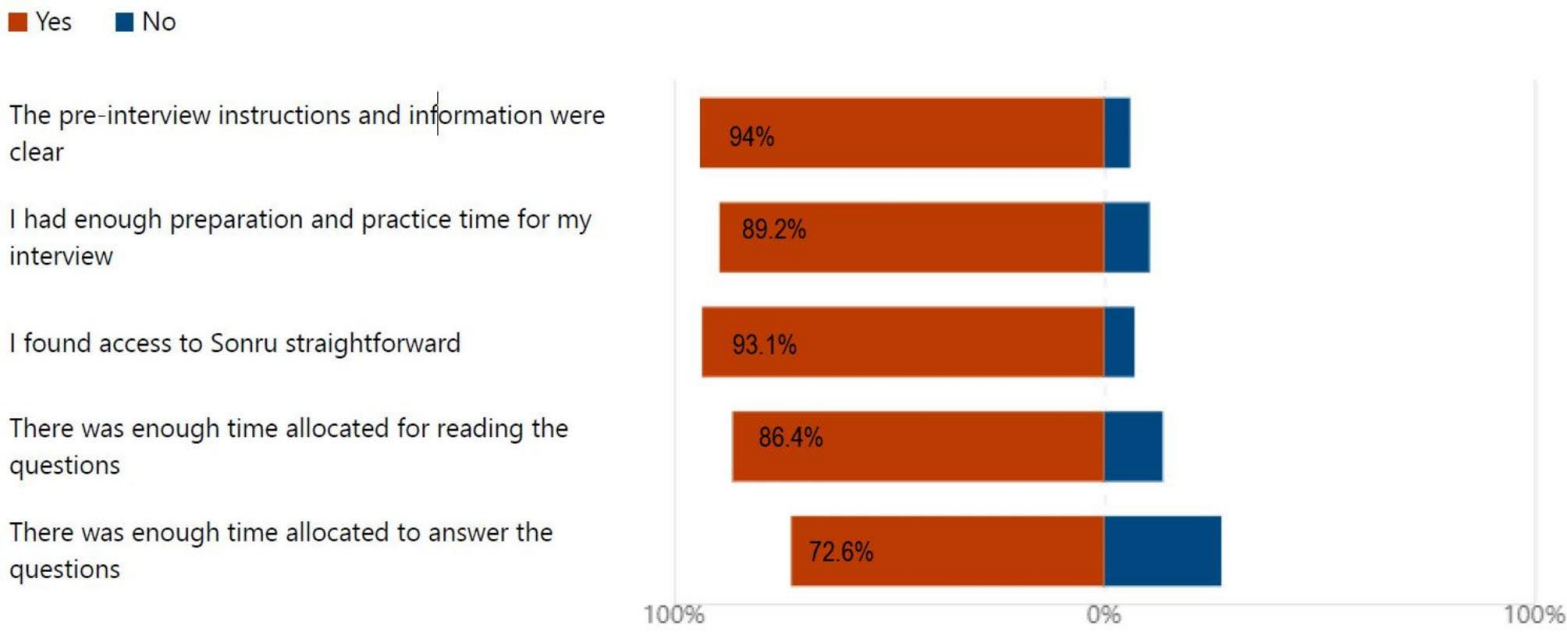
Interview instructions and Time

A total of 1569 candidates responded to the evaluation questionnaire constituting a 59% response rate. The majority were in the 17–20 age profile (n = 1028) with the 21–30 profile having 328 candidates and the remaining 213 were in the over 30 age category. The majority had expertise in digital technology as outlined in Fig. 1. The data is presented under the key questions asked:

The questions on the timing of the interview and the instructions for candidates were overwhelmingly positive with > 80% of candidates answering in the affirmative to these questions (Fig. 2).

This was reiterated again in the qualitative comments:

*I thought it was an easy process to understand, the time limits given were appropriate and I feel a 7 day deadline was great*

The flexibility around when the candidates could undertake the interview seem to be one of the reasons as to why over 80% of candidates were happy with this approach:

I am really happy with the process. I loved how I could complete the interview at a time that suited me and that I could sufficient time to prepare for it

**Fig. 3:**
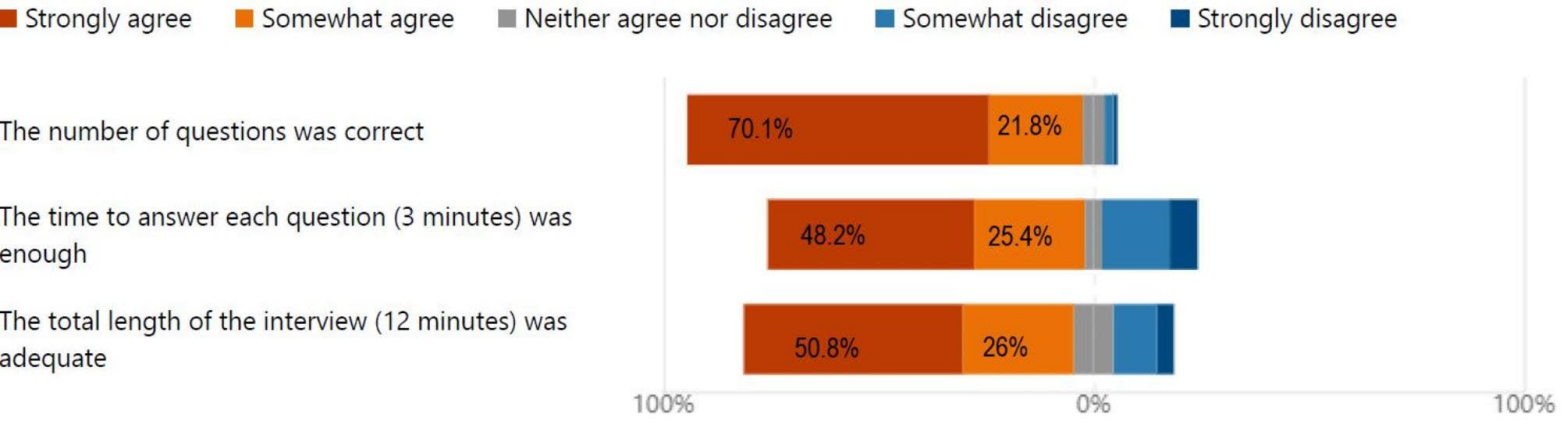
Number of questions and length of interview

Similarly, the number of questions, the time to answer and overall length of the interview generated a very positive response with 91% agreeing that the number of questions was adequate with 73.5% stating that there was sufficient time to answer the questions (Fig. 3). Additionally, 77% agreed that the length of the interview was adequate.

**Fig. 4:**
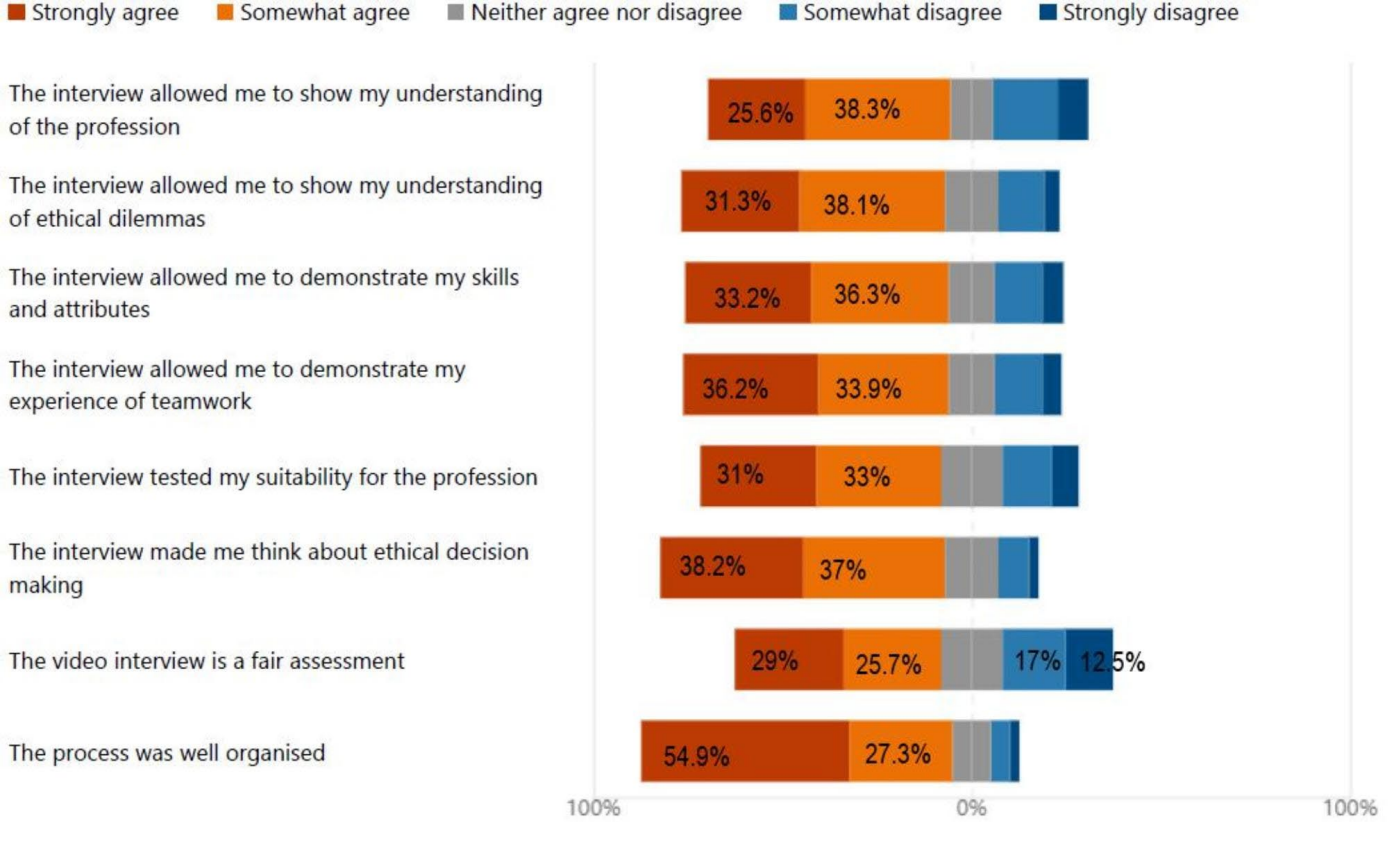
Suitability of interview format for admission to Nursing & Midwifery

Regarding the suitability and ability of the video online process enabling candidates to: demonstrate an understanding of the profession, demonstrate teamwork, discuss ethical dilemmas, show the skills and attributes required to be a nurse/midwife; all were rated positively by the candidates with over 60% of respondents either agreeing or strongly agreeing that it helped them gain an understanding of the profession as well as discuss ethical dilemmas and teamwork (Fig. 4).

*It was actually very calming and not as stressful as I expected, the questions that were asked were nice and enjoyable questions and I learnt a lot about myself while answering them*

As to whether the process was a fair assessment this was mainly positive with 55% agreeing that it was and 16% were undecided. However, 30% of respondents were less inclined to think of it as a fair assessment. This was also reflected in some of the comments;

"I did not like the video style interview and much prefer the interviews with a conversation as they are much more welcoming and not as stressful"

"I think I would have preferred a live interview but overall a good experience"

The question on the organisation of the process was evaluated exceptionally positive with 82% of candidates agreeing or strongly agreeing the process was well organised.

**Fig. 5:**
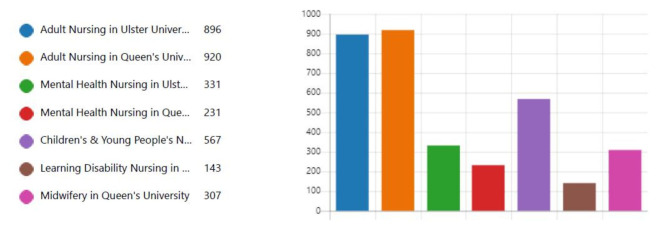
Applications to both universities. Which subject(s) did you apply for (tick all that apply)?

As was the case in previous years a significant number of candidates had applied to both universities and to a number of fields (n = 1405) (Fig. 5).

This reiterates and demonstrates the need for a more streamlined approach to admissions and supports the partnership approach. On this occasion these candidates participated in one interview whereas previously all 1405 would have been interviewed by both universities.

### The assessors

A total of 79 assessor evaluations (46%) were returned. The majority of responses were from registered nurses (n = 54); a further 7 respondents had a midwifery qualification and 7 respondents were service users. The majority of respondents had recent experience of interviewing for undergraduate nursing at either institution in the previous year; 32% (n = 25) had been working in nursing and midwifery education for less than 5 years and 30% (n = 24) more than 15 years. The majority of respondents (n = 60) were lecturers at either institution and n = 46 had been previously involved in nursing/midwifery recruitment at either institution. N = 23 respondents had no previous experience of nursing/midwifery recruitment at either institution. (The non-responders are acknowledged as a limiting factor however a 46% response rate is a credible data source).

All respondents *strongly agreed* or *agreed* that they understood the purpose of the online video interviews. While the majority strongly agreed or agreed that the online platform was explained clearly beforehand a small number were undecided (n = 6) and some disagreed/strongly disagreed (n = 3). Preparation for the role of assessor was positively rated with 92% strongly agreeing/agreeing they were adequately prepared for the role of assessor.

In relation to the interview questions 67% strongly agreed /agreed this was a relevant assessment for undergraduate entry to nursing and midwifery, while 33% strongly disagreed/disagreed or were undecided. The time allotted for assessment was very positively rated with 94% of respondents agreeing or strongly agreeing that time allotted was sufficient to allow assessment of the candidate’s performance and 92% agreeing or strongly agreeing they had sufficient time to formulate scores and write comments. 86% of respondents found the marking form clear and easy to use, with 14% (n = 11) strongly disagreeing/disagreeing.

43% (n = 34) of respondents agreed or strongly agreed that time taken to assess the candidates’ interviews was less than expected. 36% (n = 28) of respondents disagreed or strongly disagreed that the time take was less than expected and 21% (n = 17) were undecided.

The respondents were almost equally split regarding preference for interview methods; 35% of respondents rating the video-based interview as being better than the traditional interview; 33% were undecided as to a preference and 28% indicated the traditional interview using face to face formats (individual or MMI) was better. The qualitative comments help to gain a better understanding of these diverse views. The respondents who were undecided (38%) reported that they did not have experience of previous formats with which to compare, and being able to see the pros and cons in both approaches:

"I have never undertaken any interviews before this"

"No prior experience to compare"

The 35% who rated the video method as being better also reported the most common reasons for this was the flexibility it offered in viewing and scoring the video interview and more efficient use of time for the assessor. This is evidenced in their qualitative comments:

*“It is an easier process, no travel required, no other arrangements to consider, I could watch the videos in my own time at a pace I felt was right for me. I could rewind if I didn’t hear the person’s response fully”.*

*“I feel that it was an efficient use of time and you could pause, move around come back and review content if you wanted”.*

*“I really enjoyed the flexibility of the process. I also felt like I was still able to determine the candidate’s values from their answers”.*

"I think that it is possible to pick up the objectivity within the video interview which surprised me - I felt that I could definitely get a sense of the individual"

The 28% who held a preference for the traditional interview using face to face formats, individual or MMI, reported that this was better due to the opportunity for interaction and two-way communication and less chance for the candidate to read from scripts or having someone in the room helping with answers.

*“On a small number of occasions, some of the participants in the video interviews were clearly being prompted the answers in the background”.*

*“Assessing candidates in person is a better way to assess their passion through watching their interaction with actors/examiner”.*

Importantly and despite their own personal views on the system 63% (n = 50) agreed or strongly agreed the video-based interview was a fair process for applicants citing the allocation of two independent assessors as adding to the fairness of the process.

A number of areas for improvement emerged from the data. These included:

questions and marking criteria could be better aligned on Microsoft forms; improved IT connectivity and systems in place to prevent candidates reading from notes.

### Admissions

A total of six questionnaires were returned, four school based and one from each university’s central admissions teams. Regarding areas for improvement the ability to include the UCAS number was regarded as useful particularly where there were a number of applicants with the same name. Another area for improvement was the need to articulate clearly. The respondents were positive about the regional approach in general and in particular the online platform reporting that the online platform was easy to use and less complex than anticipated. One respondent cited less “clumping” of scores and the employment of a statistician as a major benefit to applicants and the advantages of sharing information across both universities to avoid numerous email queries. The allocation of videos to assessors in a more efficient manner was also raised so that the data could be returned more quickly. There was also reference to the very tight timeline for implementation of the new process and the pressures that this caused.

## Discussion

This study demonstrates the feasibility of using a regional admissions approach for selection to undergraduate nursing and midwifery programmes. By describing the development of the regional approach and tasks required in the interview process and mapping these against the values promoted by the NMC and Gateway to Nursing (NIPEC), the study provides content-related validity evidence for the interview process. Significantly the interview scores were evaluated as valid and robust and generated appropriate data to rank order and select candidates. Another important finding was the fact that 1405 candidates had made an application to both universities, thus quantifying what was known previously about duplication of workload in each university to support candidates to have two interviews. Although the overall evaluations of the new process have been essentially positive the evaluations from the key stakeholders have provided several areas for further reflection.

First evidence from the evaluations of the candidates is positive towards the new approach with many citing that they particularly liked the fact that they had only one interview to complete irrespective if they had applied to both universities. A number cited some issues with broad band connectivity. Guidance to candidates on how to manage poor network connectivity need to be fully anticipated and appropriate alternatives made available; this may include for example, guidance on how to book a computer room on a university campus.

Second, the workload for Admissions staff and school administrative staff was in some areas negatively impacted by the move to a regional process. These staff are integral to the process and it is therefore essential that concerns around how, for example, to decrease the workload by ensuring that we improve how applicants are communicated with, in particular regarding sharing information between both institutions in the partnerships are acted upon. The tight timeline for the implementation of the regional approach was also referenced as a particular challenge as admissions staff were under pressure to meet the UCAS offer date deadlines. A solution to this is to provide more realistic timelines and to anticipate and provide solutions.

Third, evaluating the robustness of the online synchronous system is important.

There were problems with some candidates’ individual computer devices and these were managed through the company provider who were able to offer technical assistance or on occasions, and in agreement with an academic member of staff, permitted the candidate to retake the interview.

Another key component to the robustness of the system is the cost-benefit analysis of the online system to previous face to face interviews. After reviewing costs i.e. administrative, academic, IT, catering, the overall savings for one institution was in the region of £30,000.

Fourth, the assessors involved in the regional approach were for the most part positive about the asynchronous nature of the interview process. A minority cited a preference for face-to-face interview; however, a number were novices to the interview process and could not therefore make a comparison across the admission methods. A limiting factor is the non-responders and any future studies should include additional follow up data collection on this particular group to ensure that all views are represented.

Finally, the implementation of a regional interview was a significant project and like all projects there will always be areas that require improvement. Selection tools for admission to nursing and midwifery are constantly under review and the selection method reported here has the potential to change how we select future students. Importantly this project provides an example of how universities can collaborate to make the admission process both effective and efficient for both the institutions and the applicants.

## Conclusion

When considering a revision to admission methods for selection to university it is important to seek the views of the key stakeholders. Our findings indicate that the move to an online interview format made the admissions process more efficient, whilst retaining a fair and transparent approach to selection. The findings indicate that an online video-based interview is a viable alternative to the more traditional face to face interview and has the potential to address the contemporary challenges in the admissions process for nursing internationally.

## Data Availability

The data sets to support this manuscript are contained within the manuscript.
